# Development of a Radiographic Index for Periodontitis

**DOI:** 10.3390/dj9020019

**Published:** 2021-02-07

**Authors:** Zeyad M. H. Shaker, Azin Parsa, Keyvan Moharamzadeh

**Affiliations:** 1 School of Clinical Dentistry, University of Sheffield, Claremont Crescent, Sheffield S10 2TA, UK; zmhshaker1@sheffield.ac.uk; 2 Adams School of Dentistry, University of North Carolina at Chapel Hill, 385 S Columbia St., Chapel Hill, NC 27599, USA; azin@live.unc.edu; 3 Hamdan Bin Mohammed College of Dental Medicine (HBMCDM), Mohammed Bin Rashid University of Medicine and Health Sciences (MBRU), Dubai 505055, United Arab Emirates

**Keywords:** radiographic index, Schei technique, panoramic radiographs, periodontitis, bone loss

## Abstract

The use of radiographic indices is noticeably diminished due to the lack of simplicity and standardisation among the existing ones. The aim of this study was to introduce a radiographic index to aid clinicians in determining the extent and severity of interproximal alveolar bone loss (iABL), in relation to individual root lengths, among patients suffering from periodontitis. A retrospective analysis of 50 anonymised dental panoramic tomograms (DPTs) of patients with periodontitis was conducted. Visual interpretation of iABL was recorded by a single investigator and by 20 volunteering clinicians for the ‘worst site’ in each quintet. Results were compared to a gold standard quantification method. Intra-examiner and inter-examiner agreement were measured using the Kappa coefficient and the intra-class correlation coefficient, respectively. Validity was assessed using Cramér’s V test. The mean intra-examiner agreement on the severity and pattern of iABL was 0.808 (_K_) and 0.802 (_K_), respectively. A stronger overall inter-examiner agreement was noted when the severity in contrast to the pattern of iABL and presence/absence of furcation involvement were analysed. The statistically significant total mean agreement values from this correlation coefficient were 0.892 and 0.739, respectively. A very strong association between all the visual interpretations carried out by all participants and the gold standard measurements was evident. Within the limitations of this study, the proposed radiographic index may serve as a simple, yet valid and reliable, adjunctive screening tool to further assist clinicians in determining the extent and severity of iABL in patients with periodontitis.

## 1. Introduction

Periodontitis is among the commonest of all forms of chronic diseases worldwide. Since a considerable loss of connective tissue and alveolar bone support via the inflammatory nature of the disease process is further complicated by an array of signs and symptoms that negatively influence the quality of life of those affected, detailed evaluation of the periodontium is a crucial element to ensure adequate patient management [1]. Thorough clinical examination and radiographic investigations are therefore critical tools used by clinicians to reach correct diagnoses, establish prognostic determinants, and formulate effective treatment plans.

In an attempt to better understand the disease process, numerous indices that chiefly rely on visual examination, periodontal probing, and/or radiographic assessments have been developed and are well documented in periodontal literature. An index system for periodontitis should be a quantitative scoring system that indicates the severity of disease with reasonable accuracy and repeatability to allow for statistical evaluation that is applicable to clinical, epidemiological, and combined clinical-epidemiological investigations [2].

Among the key advantages of developing such index systems for periodontitis was the consequent development of screening tools, such as the basic periodontal examination (BPE)—the commonly used tool in the United Kingdom since 1986. In fact, it continues to be the most popular screening assessment for indicating the level of examination required and providing basic guidance on periodontal treatment need [3].

Despite this ground-breaking benefit and the fact that several of these indices are well recognised and are used extensively in clinical and epidemiologic studies, the majority rely heavily on periodontal probing as a primary method in determining inflammation of gingival tissue and loss of periodontal attachment. Periodontal probing is inherently an imprecise technique influenced by many variables. Among the documented variables are probing techniques, the health status of the periodontal tissue, quality and precision of probe tips, local anatomical factors, length of the junctional epithelium, and presence of subgingival calculus and over-contoured restorations [4]. Thus, probing requires tactile identification of the cemento–enamel junction (CEJ), unvarying angulation and pressure with periodontal probe introduction, and accurate interpretation of the number of millimetres on the probe [5]. In addition, complaints regarding the discomfort that probing inflicts on patients should not be underestimated [6].

Whilst dental radiography is by all means not flawless, radiographic proof of alveolar bone loss has proven to be an adjunctive aid in periodontal assessment, diagnosis, treatment planning, prognostic evaluation of affected teeth, and documenting alveolar bone stability/breakdown/remodelling. Although radiographic indices, such as Sheppard’s Index (1936), Gingival-Bone Count Index by Dunning and Leach (1960), and the Bone Loss Index by Teeuw (2009) have been reportedly used in an attempt to quantify and record alveolar bone level changes among patients suffering from periodontitis, there is a noticeable absence of simplicity and standardisation that is reflected by their diminished use in population studies.

The development of a new radiographic periodontal index was thus decided, following a critical review of the existing literature. To our knowledge, there is an absence of a simple yet valid and reliable index that radiographically assesses the amount of interproximal alveolar bone loss (iABL) as a proportion of the total root length of individual teeth. Given that none of the previously developed indices used in periodontology, whether it relies on periodontal probing or radiographic quantification, is free of inherent limitations, using this proposed radiographic index in conjunction with clinical screening tools, such as the BPE, may be of great value to clinicians. Therefore, the purpose of this study was to assess the validity and reliability of the proposed radiographic index and to introduce it in an attempt to aid clinicians in determining the extent and severity of iABL in relation to root length in patients with periodontitis. It is hypothesised that this radiographic index will possibly serve as a simple yet useful adjunctive screening tool to be implemented in both clinical and epidemiological investigations.

## 2. Materials and Methods

A cross-sectional survey was undertaken to evaluate the validity and reliability of the proposed radiographic index. This involved selecting 50 anonymised dental panoramic tomograms (DPTs) taken using an Instrumentarium Dental Orthopantomograph^®^ OP200 D (Instrumentarium Dental, Nahkelantie, Finland) unit at the Radiology Department in Charles Clifford Dental Hospital (CCDH) via convenience sampling from a list of adult patients identified with a baseline diagnosis of periodontitis, who were attending for periodontal management on consultant and/or postgraduate clinics at CCDH from March 2018 to March 2019.

Following protocol review and ethics approval by The University of Sheffield, School of Clinical Dentistry (Reference No. 025305, approval date: 23 December 2019) and project authorisation by Sheffield Teaching Hospitals NHS Foundation Trust (STH20877), the 50 radiographic images were enhanced on a picture archiving and communication system (PACS) software in order to clarify alveolar bone levels and root apices of the entire dentition as much as possible and were subsequently printed using an HP LaserJet M5035xs MFP printer on Grade A Quality A4 paper upon anonymisation and standardisation to a uniform size and landscape orientation.

**Figure 1:**
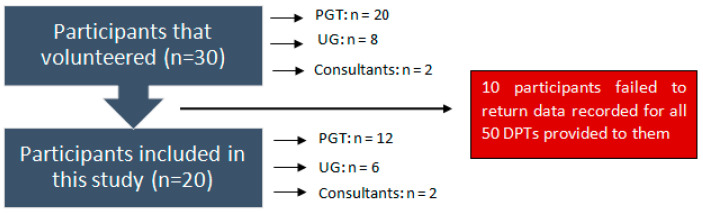
Flowchart of participants.

In order to analyse intra-examiner repeatability, a single investigator (ZS) visually interpreted the severity and extent of alveolar bone loss in relation to root lengths using the proposed radiographic index from the 50 printed DPTs in a well-illuminated room on two occasions separated by a one-month period and the results were compared. In contrast to inter-examiner repeatability, 30 volunteering participants of varying clinical experience comprised of dental undergraduate students (UG: n = 8), dental postgraduate students (PGT: n = 20), and consultants in restorative dentistry (Consultants: n = 2) were asked to visually interpret the same 50 printed DPTs and were advised to do so under adequate lighting in two equal batches separated by a one-month period. The 67% response rate allowed for the analysis of 20 participants’ visual interpretations of the 50 DPTs (Figure 1).

An appropriate sample size calculation was considered for this investigation based on data gathered from a pilot study conducted on 10 participants prior to this research. From this, a preliminary agreement was observed. Since it was hypothesised that a higher level of agreement would be achieved in this larger study, when 50 observations per subject were to be assessed and in order to achieve statistical significance for an alpha-value set at 0.05 and with the minimum power of at least 80%, the minimum number of subjects required is 19 [7] (Table 1).

An instructional presentation was delivered to the volunteering subjects detailing the aim, objectives, and methodological approach of the investigation at hand. Upon explaining the proposed scheme of scoring interproximal alveolar bone loss from DPTs, the subjects received the following:

- Participant Information Sheet;
- Consent Form;
- Instruction Sheet;
- Fifty printed DPTs (two equal batches numbered from 1–25 and 26–50, respectively);
- A Scoring Grid to fill out their recordings (to be returned to investigator ZS).

The visual interpretations (from both intra-examiner and inter-examiner data) were then compared to a gold standard alveolar bone level quantification method—Schei Technique—in order to assess the validity of the proposed index. This technique is a relative method that transforms the alveolar bone height to a fraction of the radiographic root length. A plastic ruler, ‘Schei ruler’, is used to calculate the alveolar bone level and the root length. Radiographic bone loss is considered present in sites with a cemento–enamel junction (CEJ) to alveolar crest (AC) distance greater than 1 mm. The cervical margins of any direct or indirect restorations that obscure the CEJ are used unless they are clearly apical to the expected CEJ level [8]. Thus, the following reference points are used:

- CEJ: the radiographic termination of enamel on mesial and distal aspects of the crown;
- Alveolar crest: the coronal-most level where the periodontal membrane retains its normal width—between 0.2 mm and 0.4 mm [9];
- Radiographic apex: the tip or end of the root as determined radiographically [10].

The proposed radiographic index was recorded as described below:

1. The dentition is divided into five maxillary quintets and five mandibular quintets (Table 2A);
2. The highest (worst) bone loss score for each quintet is recorded. The proposed scoring codes with additional descriptions are shown in Table 2B,C respectively;
3. All teeth in each quintet were examined (with the exception of third molars—unless first and/or second molars are missing);
4. For a quintet to qualify for recording, it must contain at least one tooth.

**Figure 2:**
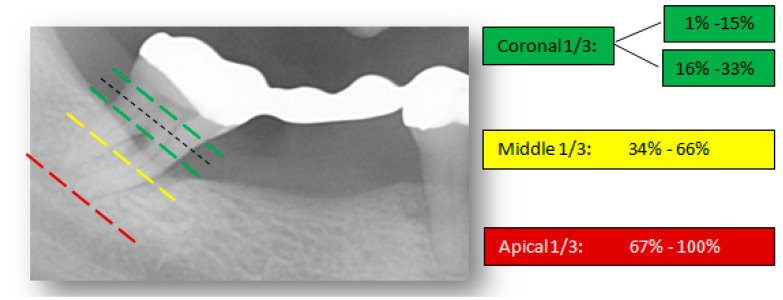
Visual interpretation of bone loss severity. The ‘worst’ tooth in each quintet is visually divided into coronal, middle, and apical thirds and the percentage of alveolar bone loss is measured accordingly.

**Figure 3:**
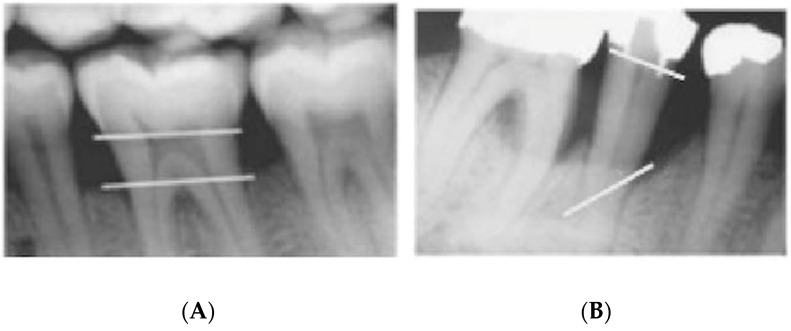
Radiographic interpretation in terms of periodontal bone loss pattern which is classified into (**A**) horizontal bone loss and (**B**) vertical bone loss.

The proposed visual and radiographic interpretation techniques to determine the severity and pattern of iABL are illustrated in Figure 2 and Figure 3.

Given the categorical nature of the data collected, an assessment of intra-examiner reliability was carried out using Cohen’s Kappa coefficient (_K_). It was the favoured measurement tool since its application accounts for the possibility of the agreement occurring by chance. The interpretation of this coefficient has been recently defined by McHugh [11].

On the other hand, to account for the potential quantitative measurement variations among the 20 examiners, the intra-class correlation coefficient (ICC) was applied to assess the consistency, reproducibility, and absolute agreement between the different groups of examiners. The proposed general guideline of ICC acceptability states that values less than 0.5 are indicative of poor reliability, values between 0.5 and 0.75 indicate moderate reliability, values between 0.75 and 0.9 indicate good reliability, and values greater than 0.90 indicate excellent reliability [12].

The validity of the proposed radiographic index was assessed using Cramér’s V test to indicate the strength of the association between the following two categorical variables with respect to the severity of iABL:

- Participants’ visual interpretations via the proposed scoring codes;
- Gold standard measurements via the Schei technique.

The interpretation of this test has been recently outlined by Akoglu [13]^.^ Cohen’s Kappa, ICC (95% confidence interval using an average-rating, absolute-agreement, two-way random-effects model) and Cramér’s V were analysed using IBM^®^ SPSS Statistics 26.0 software (IBM, Portsmouth, UK).

## 3. Results

### 3.1. Intra-Examiner Reliability

The mean intra-examiner agreement on the severity of iABL when accounting for all 10 quintets was 0.808 (_K_), which indicates a strong level of agreement. The site-specific agreements ranged between a moderate level among the lower right premolars (_K_ = 0.679) and a strong level among the lower anterior teeth (_K_ = 0.877).

The mean intra-examiner agreement on the pattern of iABL for the entire dentition and the presence/absence of furcation involvement of posterior teeth was also indicative of a strong agreement at 0.802 (_K_). In this sample, a relatively weak agreement was noted amongst the upper left molars (_K_ = 0.527), whereas a perfect agreement was evident among a few quintets, namely, the lower left premolars, lower right premolars, and lower right molars (_K_ = 1.000). These results were found to be statistically significant (*p* = 0.000) (Table 3).

### 3.2. Inter-Examiner Reliability

While considering the more experienced clinicians (PGTs and consultants in restorative dentistry), a mean inter-examiner agreement of 0.915 (ICC) was attained when the severity of iABL was assessed throughout all quintets collectively. The site-specific agreements ranged from 0.830 among the upper anteriors to 0.968 on lower right (LR) molars and 0.970 on lower left (LL) molars. When the pattern of iABL amongst all quintets and the presence/absence of furcation involvement of posterior teeth was assessed, a moderate mean inter-examiner agreement of 0.778 (ICC) was reported. These results were also found to be statistically significant (*p* = 0.000) (Table 4A).

Similarly, the dental undergraduate students showed a strong level of inter-examiner agreement (mean ICC value = 0.868) upon the assessment of the severity of iABL among all quintets. An indifferent site-specific analysis of the range was apparent in comparison to their more established colleagues. Only a moderate mean inter-examiner agreement of 0.699 (ICC) was found in this group when taking the pattern of iABL amongst all quintets and the presence/absence of furcation involvement of posterior teeth into consideration. A statistical significance (*p* = 0.000) was reached in this evaluation (Table 4B).

Upon grouping the results from all the participants included in this study, a stronger overall inter-examiner agreement was noted when the severity in contrast to the pattern of interproximal alveolar bone loss and presence/absence of furcation involvement were analysed. The statistically significant (*p* = 0.000) total mean agreement values from this correlation coefficient were 0.892 and 0.739, respectively (Table 5).

### 3.3. Validity

The results of Cramér’s V test for the association between the participants’ visual interpretations are described below.

Cramér’s V test revealed a correlation between all the visual interpretations carried out by all participants individually and the gold standard measurements. Among the 20 participants included in this study, this association ranged from 0.286 (Cramér’s V) to 0.513 (Cramér’s V). Interestingly, the weakest association came from a postgraduate dental student, whereas the strongest reported association was provided by an undergraduate dental student.

An even stronger association of 0.631 (Cramér’s V) and 0.685 (Cramér’s V) was evident among the first and second intra-examiner visual interpretations, respectively. Upon pooling these associations from intra-examiner and inter-examiner data, a statistically significant (*p* = 0.000) mean Cramér’s V of 0.447 was recorded.

## 4. Discussion

For many years, a number of limitations in current radiographic methods have been recognised, such as the two-dimensional representation of the alveolar bone and uncertainty regarding the validity, accuracy, and precision of quantitative measurements [14]. According to a recent study [15], there is a need for specific guidelines on how to estimate alveolar bone levels for calibration purposes. The authors also acknowledge the inability of dental radiographs, specifically panoramic and periapical radiographs, to accurately display bone quality and mineralisation, and to quantify alveolar bone levels circumferentially due to the lack of demonstration of alveolar bone loss in the sagittal plane.

Unfortunately, relatively few new technologies have emerged to address the critical needs in periodontal assessments. Although digital imaging has been hailed to overcome certain drawbacks, some evidence shows that the added value of digital radiography in clinical practice is mainly at a practical level and its diagnostic efficacy has in most cases been shown to be equivalent to film radiography [16]. However, more relevant research highlights that digital radiographs may in fact provide a more accurate demonstration of alveolar bone loss in contrast to their conventional counterparts [17].

In accordance with the Faculty of General Dental Practice (FGDP) guidance [18], the gold standard radiographic modalities to assess periodontal status are either a full-mouth series of periapical radiographs taken using a long-cone paralleling technique or panoramic radiographs with supplementary periapical radiographs, with the latter potentially affording a radiation dose advantage over a large number of intraoral radiographs, as well as being a quicker and less expensive option for clinicians. Additionally, several studies have demonstrated that intraoral radiographs tend to underestimate the amount of bone loss, whereas radiographic assessment of severe osseous destruction was shown to overestimate actual bone loss [19].

On the other hand, due to the limitations of panoramic radiography through the nature of the projection geometry applied, artefacts in the form of ghost and superimposed images and image magnification are frequently visible, thus essentially allowing for less image detail, especially with regards to structures outside the focal trough layer, when compared to intraoral images [20]. These inherent disadvantages may explain, for instance, the lack of clarity of anterior teeth that most participants reported on with some of the radiographic images. This feedback is corroborated by a higher number of unreadable sites due to an unidentifiable reference point(s) in the anterior and premolar quintets, which is also reflected by the relatively weaker inter-examiner agreement noticed between the observers in this investigation when visually interpreting the severity and pattern of iABL among the anterior quintets in comparison to quintets involving premolars and molars (Table 4A,B).

To test the validity of the proposed index, the optimal technique for radiographic alveolar bone level quantification required identification. Some of these quantification techniques were compared by Albandar et al. [21], by assessing detectability and readability of periapical radiographic bone level changes at baseline and after 2 years. While intra-examiner reliability was assessed and revealed similar results among all quantification techniques, inter-examiner reliability was not investigated. Regression analysis revealed that the Schei method had superior detection ability of radiographic bone level changes, while Björn’s method had the lowest detectability. As for readability, the absolute method showed the least proportion of unreadable sites in all tooth types aside from mandibular incisors where the difference between the absolute technique and the Schei technique was insignificant. The Björn method, however, indicated the highest proportion of unreadable sites. As is the case with the absolute technique, it can be inferred that, among the relative quantification techniques available, the Schei technique is accurate in assessing the radiographic changes in alveolar bone levels. These findings are further supported by a laboratory investigation carried out on dried human mandibles with the simulated progressive interdental alveolar bone loss [22].

In addition, whilst the application of the Fixot–Everett grid method to routine clinical practice seems to be more convenient in comparison to the Schei method, the former can be used only on periapical radiographs, whereas the latter method can additionally be implemented on panoramic radiographs [19].

Although a study by Teeuw et al. [23] showed promising results for alveolar bone loss detection with a novice image analyser tool when compared to a conventional method via the Schei technique, they can be explained by the slight visual impairment of the landmarks as a result of the printing process with the latter technique. Evidently, excellent inter-examiner reliability was reported between both groups. It is the authors’ opinion that the results of that study suggest that aside from the convenience factor offered by the analysing tool, there is no considerable difference between the two-bone loss calculating approaches. If more parameters, such as quantification of angular bone defects, furcation defects, and periapical radiolucencies, were incorporated in the analysing tool program, the information it would provide would be highly beneficial and would then render it more favourable than conventional techniques given that cost-effectiveness is overlooked. Indeed, it is for these evidence-based reasons that the Schei technique was favoured as the gold standard method of quantifying alveolar bone loss from panoramic radiographs in this investigation.

Moreover, dividing the dentition into five quintets per arch was proposed to improve specificity when screening for the distribution of periodontitis-affected teeth in an individual’s dentition. Unlike sextant division, it will allow for grouping similar teeth types into molars, premolars, and anteriors. The separation into quintets is hypothesised to therefore allow the molar-incisor pattern of periodontitis, especially, to be more readily detectable during the screening process. This division also allowed for separate interpretations of the results to be highlighted, such as the considerably stronger intra-examiner and inter-examiner agreements noticed among lower molars compared to upper molars with respect to the pattern and severity of iABL, respectively, which is consistent with the findings of other recent studies [24]. These observations can perhaps be explained by the relatively more complex anatomy associated with maxillary molars including the potential superimposition of palatal roots on mesiobuccal and distobuccal roots, coupled with the low negative predictive value of panoramic radiography for furcation involvement and identifying angular bone defects. In fact, the observation of an overall better agreement when the severity rather than the pattern of iABL and presence or absence of a furcation involvement were being analysed may also be influenced by these phenomena.

The percentages of iABL were purposefully selected in agreement with the severity assessment via staging in the new periodontal classification system [25]. The proposed scoring codes thus correspond to the coronal, middle, and apical thirds of a root.

## 5. Conclusions

Within the limitations of the present study, which are heavily centred on the inherent flaws of panoramic radiography, the proposed radiographic index proved to be a simple, reliable, and valid tool that may be used in conjunction with clinical screening tools to aid clinicians of varying experience levels in the prognostic evaluation of periodontally-involved teeth. Further research utilising this index on intraoral periapical radiographs would be particularly useful in supporting the findings from this project and highlighting its potential value in both clinical practice and population-based epidemiological studies.

## Figures and Tables

**Table 1:** Sample size requirement for intra-class correlation for R_0_ ≠ 0 versus R_1_ (R_0_ = 0.5 versus R_1_ = 0.7) and alpha = 0.05.

[R_0_ = 0.5] vs. [R_1_ = 0.7]		
Observation per Subject	Number of Subjects (Power = 80%)	Number of Subjects (Power = 90%)
2	63	87
3	39	55
4	32	45
5	28	40
6	26	37
7	25	35
8	24	33
9	23	32
10	22	32
20	20	28
30	19	27
40	19	27
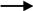 50	19	26
60	19	26
70	18	26
80	18	26
90	18	26
100	18	26

**Table 2:** (**A**) Teeth allocation in different quintets for the radiographic index. (**B**) The proposed scoring codes for the radiographic index. (**C**) The proposed additional descriptors.

(**A**)							
**UPPER RIGHT** **MOLARS** (#17–#16)	**UPPER RIGHT** **PREMOLARS** (#15–#14)		**UPPER** **ANTERIORS** (#13–#23)		**UPPER LEFT** **PREMOLARS** (#24–#25)		**UPPER LEFT** **MOLARS** (#26–#27)
**LOWER RIGHT** **MOLARS** (#47–#46)	**LOWER RIGHT** **PREMOLARS** (#45–#44)		**LOWER** **ANTERIORS** (#43–#33)		**LOWER LEFT** **PREMOLARS** (#34–#35)		**LOWER LEFT** **MOLARS** (#36–#37)
(**B**)							
**CODE**		**DEFINITION**				**PERCENTAGES (%)** **Interproximal Alveolar Bone Loss**	
**0**		No Bone Loss				0	
**1**		Mild Bone Loss				1–15	
**2**		Moderate Bone Loss				16–33	
**3**		Severe Bone Loss				34–66	
**4**		Very Severe Bone Loss				67–100	
(**C**)							
**CODE**				**DEFINITION**			
*				Furcation Involvement			
**H**				Horizontal Pattern of Bone Loss			
**V**				Vertical Pattern of Bone Loss			
**-**				Teeth Absent in Quintet			

**Table 3:** Intra-examiner agreement scores (site-specific).

Site (Quintet)	iABL Severity (_K_)	iABL Pattern/Furcation (_K_)	*p*-Value
UR Molars (1)	0.847	0.718	0.000
UR Premolars (2)	0.762	0.849	0.000
U Anteriors (3)	0.839	0.642	0.000
UL Premolars (4)	0.735	0.849	0.000
UL Molars (5)	0.844	0.527	0.000
LL Molars (6)	0.824	0.788	0.000
LL Premolars (7)	0.809	1.000	0.000
L Anteriors (8)	0.877	0.645	0.000
LR Premolars (9)	0.679	1.000	0.000
LR Molars (10)	0.862	1.000	0.000
Mean Agreement (_K_)	0.808	0.802	0.000

**Table 4:** (**A**)**.** Inter-examiner agreement scores (postgraduate students (PGTs) and consultants in restorative dentistry). (**B**). Inter-examiner agreement scores (undergraduate students (UGs)).

(**A**)			
**Site (Quintet)**	**iABL Severity (ICC)**	**iABL Pattern/Furcation (ICC)**	***p*** **-Value**
UR Molars (1)	0.932	0.847	0.000
UR Premolars (2)	0.848	0.779	0.000
U Anteriors (3)	0.830	0.480	0.000
UL Premolars (4)	0.874	0.744	0.000
UL Molars (5)	0.933	0.875	0.000
LL Molars (6)	0.970	0.906	0.000
LL Premolars (7)	0.951	0.764	0.000
L Anteriors (8)	0.907	0.731	0.000
LR Premolars (9)	0.937	0.793	0.000
LR Molars (10)	0.968	0.863	0.000
Mean Agreement ICC	0.915	0.778	0.000
(**B**)			
**Site (Quintet)**	**iABL Severity (ICC)**	**iABL Pattern/Furcation (ICC)**	***p*-Value**
UR Molars (1)	0.866	0.852	0.000
UR Premolars (2)	0.814	0.646	0.000
U Anteriors (3)	0.743	0.534	0.000
UL Premolars (4)	0.856	0.674	0.000
UL Molars (5)	0.906	0.862	0.000
LL Molars (6)	0.956	0.875	0.000
LL Premolars (7)	0.861	0.655	0.000
L Anteriors (8)	0.821	0.419	0.000
LR Premolars (9)	0.913	0.682	0.000
LR Molars (10)	0.945	0.791	0.000
Mean Agreement ICC	0.868	0.699	0.000

**Table 5:** Mean inter-examiner agreement scores for all participants (n = 20).

Participants (n = 20)	iABL Severity (ICC)	iABL Pattern/Furcation (ICC)	*p*-Value
PGT & Consultants (n = 14)	0.915	0.778	0.000
UGs (n = 6)	0.868	0.699	0.000
Total Mean Agreement ICC	0.892	0.739	0.000

## Data Availability

Not Applicable.
